# Transforming NICU care: rapid WES and transcriptomics—validation, social impact, and cost analysis

**DOI:** 10.1007/s00431-025-06225-2

**Published:** 2025-06-27

**Authors:** Beatriz Martín López-Pardo, Sofía Barbosa-Gouveia, María-Eugenia Vázquez-Mosquera, Francisco Reyes, Claudia Falcão Reis, Francisco Laranjeira, Tomas Sánchez-Tamayo, Paula Sánchez-Pintos, Cristina Durán Fernández-Feijoo, Alejandro Pérez-Muñuzuri, María-Luz Couce

**Affiliations:** 1 Unit of Diagnosis and Treatment of Congenital Metabolic Diseases, Department of Neonatology, Santiago de Compostela University Clinical Hospital, Choupana Street, 15704 Santiago de Compostela, Spain; 2 IDIS-Health Research Institute of Santiago de Compostela, Santiago de Compostela University Clinical Hospital, Choupana Street, 15704 Santiago de Compostela, Spain; 3 European Reference Network for Hereditary Metabolic Disorders (MetabERN), Santiago de Compostela University Clinical Hospital, Choupana Street, 15704 Santiago de Compostela, Spain; 4 Faculty of Medicine, University of Santiago de Compostela, Rúa de San Francisco, s/n, 15782 Santiago de Compostela, A Coruña Spain; 5 Centro de Investigación Biomédica en Red de Enfermedades Raras (CIBERER), Monforte de Lemos Street, 3-5, 28029 Madrid, Spain; 6 Red RICORS-SAMID, ISCIII, Madrid, Spain; 7 Centro de Investigaciones en Red en Enfermedades Cardiovasculares (CIBERCV), Monforte de Lemos, 3-5, 28029 Madrid, Spain; 8 Unit of Medical Genetics, Jacinto Magalhães Medical Genetics Center, Centro Hospitalar Universitário de Santo António, Largo Prof. Abel Salazar, 4099-001 Porto, Portugal; 9 Multidisciplinary Unit for Biomedical Research (UMIB), Institute of Biomedical Sciences Abel Salazar (ICBAS), University of Porto, Jorge Viterbo Ferreira Street, 228, 4050-313 Porto, Portugal; 10 School of Medicine, Life and Health Sciences Research Institute (ICVS), University of Minho, Gualtar Campus, 4710-057 Braga, Portugal; 11 ITR - Laboratory for Integrative and Translational Research in Population Health, Jorge Viterbo Ferreira Street, 228, 4050-313 Porto, Portugal; 12 Department of Pediatrics, Hospital Regional Universitario de Málaga, Avenida de Carlos Haya, S/N, 29010 Málaga, Spain

**Keywords:** Cost analysis, Neonatal genomics, RNA analysis, Neonatal Intensive Care, Parenting Stress Index

## Abstract

Genetic diseases significantly contribute to morbidity and mortality in neonatal intensive care units (NICUs), with diagnoses often delayed due to clinical complexity. Rapid whole-exome sequencing (rWES) and transcriptomic analysis (RNA-seq) may improve diagnostic rates and clinical outcomes. Prospective study of neonates admitted to NICUs with suspected genetic diseases (*n* = 34) who underwent rWES, followed by RNA-seq applied in cases in which rWES failed to establish diagnosis. The primary outcome was the diagnostic rate. Secondary outcomes included time to diagnosis, clinical utility, parental stress, and cost-effectiveness. rWES achieved a 41% diagnostic rate with a mean turnaround time of 8.57 ± 2.62 days. RNA-seq increased the diagnostic yield by 6%, resulting in a total diagnostic rate of 47%. The use of rWES reduced unnecessary procedures by 15% and shortened hospital stays by 25% (*p* < 0.01). Cost-effectiveness analysis indicated that rWES was economically advantageous, with an ICER of < €9000. Relative to pre-diagnosis levels, parental anxiety decreased by 30% in cases in which diagnosis was achieved but increased by 15% in cases in which no diagnosis was established (*p* < 0.05).

*Conclusion*: Implementing rWES in NICUs improves care for critically ill neonates by providing timely, accurate diagnosis, reducing healthcare costs, and alleviating parental anxiety. RNA-seq further enhances diagnostic accuracy.

**Graphical Abstract:**

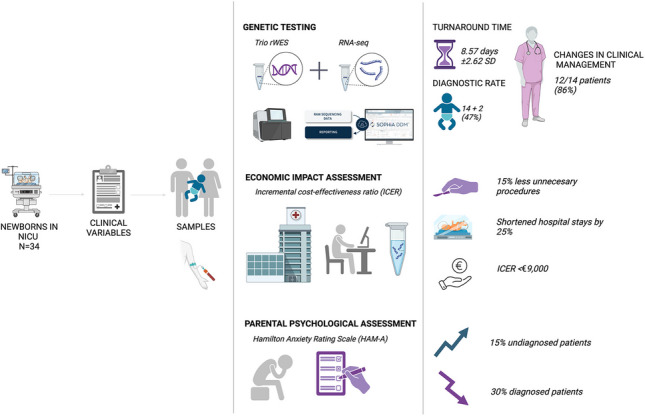

**Supplementary Information:**

The online version contains supplementary material available at 10.1007/s00431-025-06225-2.

## Introduction

Genetic alterations are a major cause of morbidity and mortality in neonatal intensive care units (NICUs). Clinical heterogeneity and overlapping symptoms complicate diagnosis, which is often delayed or established post-mortem [1, 2]. Given the potential for rapid disease progression and high lethality in the vulnerable newborn population, diagnosis and timely intervention are vital for critically ill patients [3]. Genomic sequencing, including whole-exome sequencing (WES), has evolved from research-specific technologies to tools that are increasingly integrated into clinical practice [4, 5]. Several studies have demonstrated the validity of rapid whole-exome sequencing (rWES) as a first-tier approach to diagnosis in patients admitted to NICU, with diagnostic rates ranging from 30 to 50%, and a mean time to diagnosis of 11–16 days [6–8]. Trio exome sequencing, which includes both parents and the proband, further reduces turnaround time (TAT) and maximizes diagnostic accuracy [9, 10] About 90% of rWES-related health savings come from shorter hospital stays and fewer major procedures, while 10% of results from avoided diagnostic tests, like microarrays [11]. RNA-seq has been proposed to improve genetic diagnosis, especially for rare diseases [12]. Transcriptomics remains unevaluated in critically ill neonates but can enhance genetic diagnosis, increasing diagnostic rates and benefiting both research and clinical practice [13]. Moreover, few studies have investigated trio-based genetic testing in the neonatal period, highlighting a significant research gap that needs to be addressed [14, 15].

This study assesses rWES diagnostic efficacy and introduces RNA-seq in critically ill newborns with suspected genetic disorders. It also examines the psychosocial impact of non-diagnosis and the cost-effectiveness of its integration into NICU protocols.

## Methods

### Study design

This multicentre prospective study included 34 patients admitted to NICUs in four tertiary hospitals in Spain and Portugal over a 24-month period. Participants were recruited through the Santiago Clinical University Hospital, the Malaga Regional Hospital and the Miguel Servet Clinical University Hospital, in Spain and the Centro Hospitalar do Porto, in Portugal. Written informed consent was obtained from the patient’s parents or legal guardians prior to their inclusion. The study was approved by the Ethics and Clinical Research Committee of Galicia (registry code 2020/494) and conducted in accordance with the principles of the Declaration of Helsinki.

### Study population

Patients under 2 months of age were included if they met at least one of the following criteria: (i) congenital malformations not attributable to a defined genetic syndrome; (ii) clinical signs of metabolic decompensation in the absence of biochemical abnormalities and/or neonatal screening findings suggestive of an inborn error of metabolism; (iii) epilepsy or neurodevelopmental disorders with suspected genetic origin based on family history, consanguinity, or clinical presentation; (iv) severe encephalopathy or hypotonia of unknown cause; (v) respiratory or cardiovascular failure without acquired explanations and with features suggesting a genetic basis (such as multisystem involvement or positive family history); or (vi) other findings indicative of an underlying genetic disorder, including dysmorphic features, multisystem involvement, or an unexplained severe disease course.

### Study variables

Included family history, gestational age, clinical signs, days since NICU admission, laboratory and imaging results, treatments, and mortality. Clinical procedures performed before and after genetic diagnosis in the trio were documented to assess clinical utility and economic impact. Transcriptomic analysis introduced a new dimension that increased diagnostic accuracy, leading to an improved diagnosis rate. Phenotypic features were recorded using Human Phenotype Ontology (HPO) terms. Differential diagnoses were generated using the Phenomizer tool (http://compbio.charite.de/phenomizer/). Clinical and molecular findings were then correlated with genetic study results.

### Procedures

#### Blood collection from patient and parents

A 3 ml blood sample was collected from each trio (patient and both parents) for DNA extraction. For patients in whom rWES failed to establish a diagnosis, 2 ml blood samples were collected in Paxgene tubes for RNA extraction.

#### Genetic testing

rWES was performed using the Human Core Exome Kit (Twist Bioscience), and sequencing was performed on an Illumina NextSeq platform. Raw data were analyzed using the SOPHiA DDM® platform, following American College of Medical Genetics guidelines for variant classification.

#### RNAseq

RNA was processed according to the manufacturer’s recommendations (Illumina, San Diego, CA, USA). Libraries were prepared from total RNA using the TruSeq Stranded mRNA Library prep kit (Illumina). RNA integrity and concentration were determined by Nanodrop using the Qubit RNA Broad-Range Assay. All libraries achieved an average size range of 300 bp. An Agilent 4200 Tapestation RNA Screentape was used to confirm library quality by RNA size analysis. Paired-end, 150-cycle sequencing was performed on the 500/550 High Output NextSeq platform (Illumina). The Genome Analysis Toolkit (GATK, v4.2.4.1) Best Practices workflow was used for variant calling. The resulting variant call format files were further annotated using Variant Effect Predictor (VEP, v.104) to identify clinically relevant variants.

### Differential expression analysis and aberrant splicing

Analysis of aberrant expression was performed using OUTRIDER [16] and aberrant splicing was analyzed using FRASER2 [17]. OUTRIDER was used to detect outliers in gene expression by fitting a robust statistical model to the RNA-seq count data, while FRASER2 identified aberrant splicing events by analyzing splicing junction counts and detecting splicing outliers in the RNA-seq data. For differential expression analysis, read counts for each gene and transcript were quantified using RNA-SeQC (v. 2.4.2) [18]. The resulting count matrix was then imported into DESeq2 (v. 1.44) for normalization and differential expression testing [19]. DESeq2 uses a model based on the negative binomial distribution to identify differentially expressed genes (DEGs) between conditions. Significantly, differentially expressed genes were identified using an adjusted *p*-value threshold (FDR < 0.05) and a log2 fold change cut-off of ± 1.

### Economic impact assessment

The economic impact on the Spanish National Health Service was assessed by comparing the costs of rWES with the costs of delayed diagnosis. These included direct healthcare costs as well as indirect costs. Cost estimates are from Galician health service tariffs [20] and included the cost of the average 6-year diagnostic delay typical for rare diseases [21]. Calculation of the cost of metabolic testing included consumables, personnel, and equipment amortization (totaling €1856.8 per test) [21]. Non-healthcare-related costs that were considered included parents’ loss of earnings (€34,670) [22] based on the average gross income of a worker (€20,288.1) [23], as well as lost leisure time, calculated at 47% of working hours [24]. The incremental cost-effectiveness ratio (ICER) was used [25] to compare the cost of rWES with costs resulting from delayed diagnosis. Two measures were used to assess the effectiveness of rWES: (i) reduction in parental anxiety, using the Hamilton Anxiety Rating Scale (HAM-A) before and after receiving genetic results; and (ii) impact on mortality, calculated as the difference in probability of dying with and without genetic diagnosis.

### Parental psychological assessment

The clinical impact of the diagnosis was assessed using the HAM-A test, which measures family anxiety. The clinician performed this test twice: before and after the family received the genetic results, positive or negative.

### Statistical analysis

Categorical variables are presented as percentages, and continuous variables as mean ± standard deviation (SD) or median and interquartile range for variables with a non-normal distribution. Phenomizer was used to identify candidate diseases based on the clinical characteristics of each patient. *p*-values were estimated by Monte Carlo random sampling and corrected for multiple testing using the Benjamini and Hochberg method. A *p*-value < 0.05 was considered statistically significant. Mean HAM-A test scores were compared using the non-parametric Mann–Whitney test.

## Results

### WES results

During the study period (July 2021 to June 2023), a total of approximately 3125 NICU admissions occurred across the four participating tertiary hospitals. Among these, 55 newborns were admitted with suspected genetic conditions: 664 at Santiago Clinical University Hospital, 846 at Miguel Servet Clinical University Hospital, 832 at Malaga Regional Hospital, and 783 at Centro Hospitalar do Porto. Twelve patients were diagnosed prenatally or at birth and were therefore excluded. In six cases, parental genetic testing was not available, and in three, informed consent was not obtained. The final cohort comprised 34 newborns who underwent rWES (Online Resource 1), with a median enrolment age of 4 days.

**Fig. 1:**
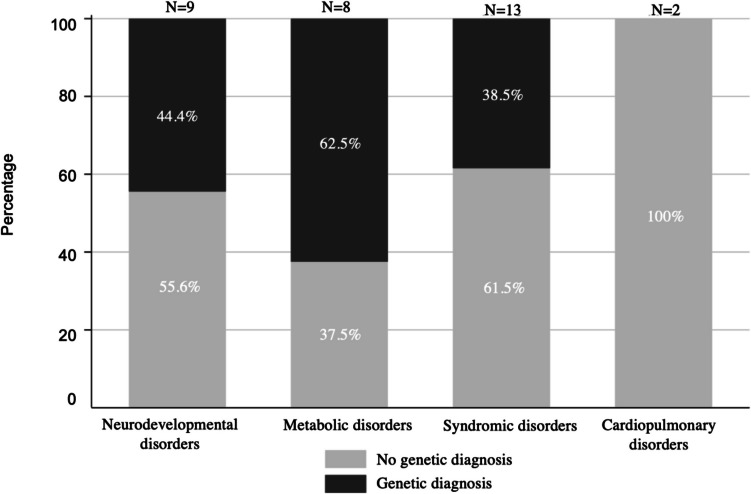
Percentage of patients diagnosed according to type of disorder. Chart depicts the proportions of each genetic disorder type in the study population, and the proportion of neonates within each category for whom a diagnosis was established

Implementation of rWES allowed rapid molecular diagnosis, with a mean time to diagnosis of 8.57 days ± 2.62 SD in 14 patients, as well as early and appropriate clinical action and initiation of therapeutic support. Characteristics and genetic findings of patients with a confirmed diagnosis through rWES are presented in Table 1. This table provides comprehensive data, including identified genes, specific variants, inheritance patterns, and associated phenotypes. A diagnostic rate of 41% was achieved, identifying neurodevelopmental, metabolic, and syndromic disorders (Fig. 1). Neurodevelopmental disorders included familial benign neonatal epilepsy (OMIM#121200), developmental and epileptic encephalopathy 68 (OMIM#618367), and spastic paraplegia 91 (OMIM#616670), with two patients diagnosed with familial benign neonatal epilepsy (OMIM#121200). Metabolic disorders comprised Niemann-Pick type C disease (OMIM#257220), Wolman disease (OMIM#278000), sulfite oxidase deficiency (OMIM#272300), transient infantile liver failure (OMIM#613070), and combined oxidative phosphorylation deficiency 3 (OMIM#610505). Syndromic disorders included CHARGE syndrome (OMIM#214800), Kabuki syndrome 1 (OMIM#147920), MIRAGE syndrome (OMIM#617053), Waardenburg syndrome (OMIM#193500), and polycystic kidney disease 4 (OMIM#263200). Phenomizer analysis provided a differential diagnosis with *p* < 0.05 in five patients and *p* = 0.05 in three. In two cases, the *p*-value was unavailable as the suspected disease was not in the Phenomizer database.

**Table 1:** Clinical data and genetic findings for each patient for whom rWES yielded a positive diagnosis

Cases	Age	Sex	HPO	Gene	Variants	ACMG classification	Phenomizer	Disorder	OMIM	Parents/inheritance
1	2 d	M	Lethargy Spastic tetraparesis Subdural hematoma Mild ventriculomegaly Bilateral talipes equinovarus	*SPTAN1*	NM_001130438.2: c.6910_6918 dupCAGCTGGGC	LP	-	Spastic paraplegia 91	#620538	De novo Autosomal dominant
2	2 d	F	Neonatal intestinal obstruction Sensorineural hearing impairment Aganglionic megacolon (Hirschprung disease)	*SOX10*	NM_006941.3: c.850G > T	P	*p* = 0.11	PCWH syndrome	#609136	De novo Autosomal dominant
3^†^	22 d	M	Hepatomegaly Liver failure Neonatal jaundice	*NPC1*	NM_000271.4: c.2612 A > G/c.3662 del	P LP	*p* = 0.001	Niemann-Pick disease	#257220	Carriers Autosomal recessive
4	3 d	F	Coloboma Optic atrophy Strabismus Visual impairment Mixed hearing impairment Septal defect	*CHD7*	NM_017780.3: c.5458 C > T	P	*p* = 0.05	CHARGE syndrome	#214800	De novo Autosomal dominant
5	1 d	F	Hyponatremia Ascending aortic dilation Left ventricular hypertrophy Polycystic kidney dysplasia Enlarged kidneys Respiratory distress	*PKHD1*	NM_138694.3: c.2269 A > C/c.107 C > T	VUS/P P	*p* = 0.05	Polycystic kidney disease 4, with or without hepatic disease	#263200	Carriers Autosomal recessive
6	1 d	F	Lactic acidosis Feeding difficulties Respiratory failure Intrauterine growth retardation	*TSFM*	NM_001172696.2: c.997 C > T/c.997 C > T	P	*p* = 0.028	Combined oxidative phosphorylation deficiency 3	#610505	Carriers Autosomal recessive
7	1 d	F	Focal seizures Generalized seizures	*KCNQ2*	NM_004518.6: c.1603G > A	P	*p* = 0.006	Seizures, benign neonatal	#121200	De novo Autosomal dominant
8	20 d	F	Hypertriglyceridemia Elevated hepatic transaminases Hepatomegaly Splenomegaly	*LIPA*	NM_000235.3: c.966 + 2 T > G/c.966 + 2 T > G	P	*p* = 0.053	Wolman disease	#620151	Carriers Autosomal recessive
9	2 d	F	Hemolytic anemia Abnormal face shape Atrial septal defect Ventricular septal defect Abnormality of the outer ear Generalized hypotonia Muscular hypotonia Encephalopathy Abnormality of the vertebrae Congenital hip dislocation	*KMT2D*	NM_003482.3: c.10229 del	P	*p* = 1.000	Kabuki syndrome	#147920	De novo Autosomal dominant
10	1 d	F	Thrombocytopenia Adrenal insufficiency Recurrent infections	*SAMD9*	NM_017654.3: c.2945G > A	LP	*p* = 0.62	MIRAGE syndrome	#617053	De novo Autosomal dominant
11	1 d	F	Focal seizures Generalized seizures	*KCNQ2*	NM_004518.5: c.242 T > C	VUS/LP	*p* = 0.006	Seizures, benign neonatal, 1	#121200	Maternal (symptomatic) Autosomal dominant
12^†^	1 d	F	Lactic acidosis Encephalopathy Generalized seizures	*SUOX*	NM_001032386.1: c.905 T > G/c.905 T > G	P	*p* = 1.22	Sulfite oxidase deficiency	#272300	Carriers Autosomal recessive
13^†^	1 d	M	Increased serum lactate Lactic acidosis Feeding difficulties Hepatomegaly Jaundice Muscular hypotonia	*TRMU*	NM_018006.4: c.1258_1259 dup/c.1258_1259 dup	LP	*p* = 0.003	Liver failure	#613070	Carriers Autosomal recessive
14	1 d	M	Generalized tonic–clonic seizures	*TRAK1*	NM_001042646.2: c.994 A > G/c.1957 A > T	VUS/LP VUS	-	Developmental and epileptic encephalopathy, 68	#618201	Carriers Autosomal recessive

^†^Deceased

*d*, day; *F*, female; *HPO*, Human Phenotype Ontology; *LP*, likely pathogenic; *M*, male; *P*, pathogenic; VUS, variant of uncertain significance; *WES*, rapid whole exome sequencing

In patients 15 and 16, the initial rWES analysis was negative. However, given their parents’ interest in future pregnancies, further molecular studies were conducted externally, leading to a confirmed genetic diagnosis. Patient 15 was found to carry a homozygous intronic variant classified as a variant of uncertain significance (VUS). While not located within a canonical splice site, intronic positions near exons can affect splicing regulatory elements, making experimental validation necessary to determine its impact. In patient 16, a likely pathogenic missense variant was identified along with a deletion of exons 8 and 9. Both variants were in *NARS2*, associated with combined oxidative phosphorylation deficiency (OMIM#616239). Tragically, both patients passed away within the first months of life.

In 12 of 14 diagnosed newborns (86%), rWES led to management changes within three months. Medication was adjusted in 8 cases (57%), including 2 (14%) with targeted treatments. Intensive care was withdrawn for 6 patients (43%), initiating palliative care. Eight (57%) received new subspecialty care, and all families received prompt genetic counseling.

### Transcriptomic analysis

**Fig. 2:**
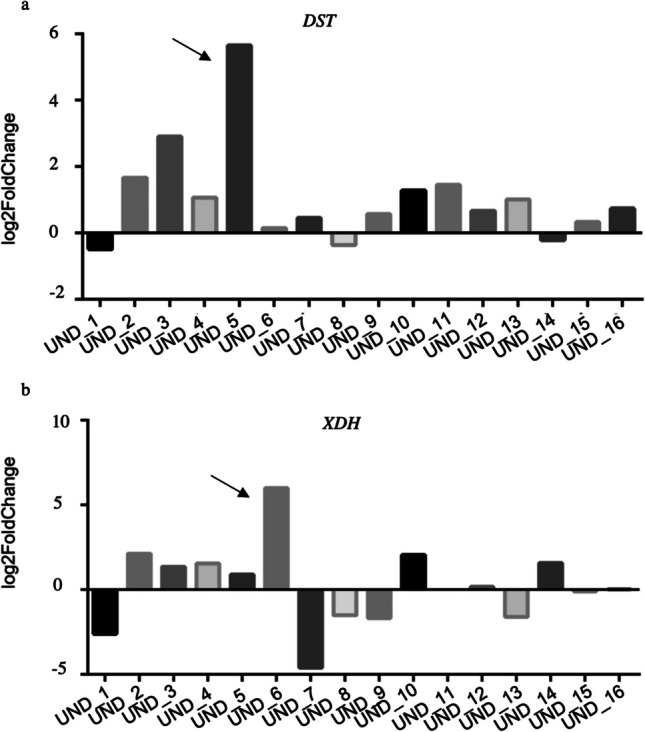
Differential gene expression as determined by RNA-seq analysis. Gene expression rate for *DST* (Fig. 3a) and *XFH* genes (Fig. 3b). Black arrow highlights the two possible diagnoses in patients UND_5 and UND_6

rWES failed to diagnose 18 of 34 neonates. Paxgene samples were unavailable for 2 cases, so RNA-seq was performed on the remaining 16 UND patients. Gene expression differences in 2 cases raised the diagnostic rate to 47%. UND_5 (26 days) had axial hypotonia, bilateral clubfoot, and hand rotation with abduction since birth. The patient required respiratory support until day 15 and had poor sucking reflexes. RNA-seq found no variants or aberrant splicing but detected DST gene expression changes linked to hereditary sensory neuropathy type VI (OMIM #614653). In UND_6 (15 days), with mild xanthinuria, low uric acid, and hyperglycinuria, RNA-seq revealed XDH expression changes associated with xanthinuria type I (OMIM #278300) (Fig. 2). All libraries had an average size of 300 bp.

The diagnostic rate using rWES was 41%, with an additional 6% of cases successfully diagnosed after the initial rWES analysis and a further 6% identified by RNA-seq, for a total diagnostic rate of 53% (Table 2).

**Table 2:** Clinical data and genetic findings for patients for whom a positive diagnosis was established after negative rWES results (15 and 16) and patients with possible molecular diagnosis via RNA-Seq analysis

Cases	Age	Sex	HPO	Gene	Variants	ACMG classification	Phenomizer	Disorder	OMIM	Parents/inheritance
15^†^	1 d	F	Pulmonary hypoplasia Persistent pulmonary hypertension Arterial hypotension Hyperglycemia	*NARS2*	NM_024678.6: c.959 + 9 A > G/c.959 + 9 A > G	VUS VUS	*p* = 1.00	Combined oxidative phosphorylation deficiency 24	#616239	Carriers Autosomal recessive
16^†^	1 d	F	Hypotonia Lactic acidosis Ventricular septal defect Intrauterine growth retardation Premature birth	*NARS2*	NM_024678.6: c.500 A > G/c.(822 + 1_8231)_(959 + 1_960-1)del(p.?)	P LP	*p* = 0.395	Combined oxidative phosphorylation deficiency 24	#616239	Carriers Autosomal recessive
UND_5	26 d	M	Axial hypotonia Bilateral talipes equinovarus Respiratory insufficiency	*DST*	-		*p* = 1.00	Hereditary sensorial neuropathy, type VI	#614653	-
UND_6	15 d	F	Xanthinuria Abnormality of urinary uric acid concentration Hyperglycinuria	*XDH*			*p* = 0.1277	Xanthinuria, type I	#278300	-

^†^Deceased

*d*, day; *F*, female; *HPO*, Human Phenotype Ontology; *LP*, likely pathogenic; *M*, male; *P*, pathogenic; *VUS*, variant of uncertain significance; *WES*, rapid whole exome sequencing

### Economic impact assessment

Table 3 shows the results of the cost-effectiveness analysis of rWES as a diagnostic tool, comparing the cost of implementation with the estimated costs associated with delayed diagnosis. To understand these results, we must compare them with the available thresholds. Thus, we applied the thresholds proposed by Pinto Prades [24], who established a rWES cost range of − €9000 to €42,000. As shown in Table 3, the cost of rWES was effective in all cases when we analyzed attending costs and beneficial unit prices (ICER value ≤ the proposed comparison of €9000). Then, we calculated cost-effectiveness according to the probability of mortality. Online Resource 2 shows the results for cost-effectiveness measured as the probability of mortality in early versus late diagnosis scenarios. We use the same comparator as described by Pinto Prades [26] and an established rate of €42,000. The results show that achieving an early diagnosis by WES is cost-effective for all disorders detected in our cohort, except for familial benign neonatal epilepsy.

**Table 3:** Cost-effectiveness analysis: cost of using rWES as a diagnostic tool versus the inferred costs associated with delayed diagnosis (ICER A) and described cases in which the health cost is effective when we analyze attending the costs and beneficial unit prices (ICER B)

Disorder	ICER A (€)	ICER B (€)
Niemman-Pick type C	1488.01	12,549.85
CHARGE syndrome	3720.02	35,428.77
Combined oxidative phosphorylation deficiency 3	1488.01	10,054.11
Waardenburg syndrome, type 2E, with or without neurologic involvement	744.00	-
MIRAGE syndrome	− 7440.04	-
Sulfite oxidase deficiency	1860.01	-
Transient infantile liver failure	− 3720.02	22,044.57
Wolman disease	1860.01	12,400.07
Polycystic kidney disease 4 with or without polycystic liver disease	− 676.37	21,257.26
Seizures, benign neonatal, 1	− 1488.01	46,500.26
Developmental and epileptic encephalopathy 5	− 7440.04	-
Developmental epileptic encephalopathy 68	− 1240.01	-
Kabuki syndrome	1860.01	-

*ICER*, incremental cost-effectiveness ratio

### Psychological assessment

**Fig. 3:**
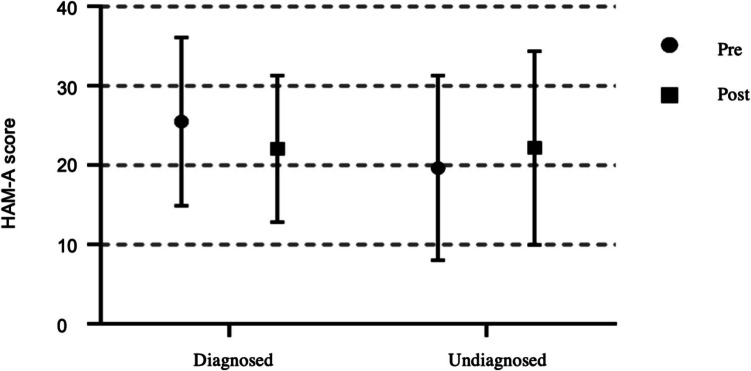
Impact of genetic diagnosis on parental anxiety levels. Figure shows Hamilton Anxiety Rating Scale (HAM-A) scores for the parents of neonates in the study, comparing anxiety levels before (pre) and after (post) the results of the genetic analysis were provided, stratified according to test result (diagnosed or undiagnosed)

Parental anxiety, measured using the HAM-A scale, decreased by 30% after receiving a definitive diagnosis, but increased by 15% in cases without a diagnosis (*p* < 0.05) (Fig. 3). Families with a confirmed result expressed relief from uncertainty and greater emotional stability, while those without a diagnosis reported increased distress due to the lack of answers. These results highlight the emotional value of achieving a genetic diagnosis and the need for psychological support when no clear outcome is reached.

## Discussion

WES is a powerful diagnostic tool for NICU neonates, with rWES delivering results in days, allowing timely interventions and informed care decisions, including comfort-focused care when treatment is limited. The diagnostic efficacy of WES and WGS in critically ill infants has been described in previous studies, with reported rates of 31% (WGS) [27], 41–47% (WES) [28, 29], and 35–37% (rWES) [11, 30]*.* D’Gama et al. applied rWES in the NICU as part of a pilot research study and reported a diagnostic yield of approximately 35% with a time to diagnosis of 13–20 days in a cohort of critically ill infants [30]. Adhikari et al. reported that WES had a sensitivity of 88% and a specificity of 98.4%, compared to 99% and 99.8% for biochemical screening [31]. The authors concluded that while WES is valuable, it may not be suitable as a stand-alone primary test for certain conditions, but can effectively reduce false positives when used as a second-line test. Kingsmore et al. reported a mean diagnostic yield of approximately 37% with a mean TAT of 11 days, based on a combination of 44 studies of children in ICUs with diseases of unknown etiology [11]. All the studies highlight the effectiveness of rWES in providing timely and accurate diagnoses in urgent clinical scenarios. In our study, a definitive molecular diagnosis was established rapidly (mean, 8.5 [range: 7–10] days) compared to previous studies, with an rWES diagnostic rate of 41%, demonstrating the effectiveness, robustness, and clinical relevance of our diagnostic approach. Moreover, we showed that RNA-seq complements rWES by identifying gene expression anomalies, such as aberrant splicing or expression, that may not be captured by WES alone. The application of RNA-seq in our cohort increased our global diagnostic rate by a further 6%. These findings underscore the importance of rapid diagnostic techniques in enhancing clinical care for neonates with genetic disorders.

The ability of rWES to provide rapid and comprehensive genetic insights is crucial for the clinical management. Early and accurate diagnosis can significantly influence treatment decisions, allowing for tailored therapeutic interventions that can improve patient outcomes. Stark et al. emphasized the role of rWES in facilitating precision medicine approaches by identifying actionable genetic variants that guide specific treatments [29]. Early targeted therapy can determine prognosis, preventing inappropriate treatments and guiding decisions in critical cases. One patient in our study was diagnosed with Wolman disease, a rare lysosomal disorder. Previously, hematopoietic stem cell transplantation (HSCT) was the only treatment, but its complexity and risks often led to post-transplant liver failure and death [32]. Advances in diagnostics now enable earlier detection and the adoption of new therapies, such as Sebelipase alfa, which has replaced HSCT as a life-saving alternative [33]. Our Wolman patient, now 3 years old, receives weekly Sebelipase alfa and follows a low-lipid diet, leading a normal life [34]. This case highlights the impact of early therapy on prognosis and quality of life. In cases without optimal treatments, decisions should prioritize patient well-being and caregiver support. One patient was diagnosed with combined oxidative phosphorylation deficiency 3 (OMIM#610505), a rare mitochondrial disorder with variable expression, lactic acidosis, hypotonia, rhabdomyolysis, seizures, and early progressive encephalopathy, often leading to high mortality within months [35]. Knowing the fatal prognosis allowed healthcare providers to prepare the family and initiate end-of-life care, ensuring the patient’s well-being and dignity through family involvement.

The implementation of rWES provided families with crucial genetic counseling, reducing anxiety and supporting informed decision-making. Prior studies highlight that rapid genetic diagnoses alleviate parental distress by clarifying prognosis and guiding medical decisions [27, 36]. Clark et al. [27] reported that early molecular diagnoses help parents understand and manage their child’s illness, while genetic counseling mitigates uncertainty. Consistent with this, our study found high pre-test HAM-A anxiety scores, which significantly decreased in parents who received a diagnosis, reinforcing previous findings on the psychological benefits of genetic testing. In contrast, undiagnosed cases showed increased anxiety, underscoring the emotional burden of uncertainty. Receiving a clear diagnosis reduces parental anxiety, demonstrating the dual clinical and emotional benefits of genetic testing [36]. These findings emphasize the need for additional psychological support and genetic counseling when a diagnosis is not established.

The present study is the first to evaluate the economic impact on the Spanish National Health System of using this strategy in patients admitted to the NICU. The ability of rWES to streamline the diagnostic process and reduce the need for multiple sequential tests may lead to overall cost savings for healthcare systems. Our results show that the use of rWES to achieve early diagnosis is cost-effective for all conditions detected in our cohort except familial benign neonatal epilepsy. rWES demonstrated high cost-effectiveness not only in reducing unnecessary diagnostic tests but also in shortening hospital stays. Sanford Kobayashi et al. also found that rWES reduced the length of hospital stay and avoided unnecessary diagnostic tests and procedures, and attributed the savings mainly to early and accurate diagnosis, which allowed for targeted treatments and interventions [37].

There are certain limitations associated with the use of rWES in the NICU related to the interpretation of variants of uncertain significance (VUS) and the possibility of misdiagnosis due to technical limitations or sample quality issues. CNV detection in rWES with a limited sample size is challenging due to the reliance on depth-of-coverage analysis, which requires a robust reference set for normalization. This reduces statistical power, making it difficult to distinguish true CNVs from sequencing variability. Additionally, rWES prioritizes speed over deep coverage, in order to get a faster diagnosis in most of the cases, although, with further limiting CNV resolution. In this sense, CNVs could not be detected in our cohort. However, as often occurs with negative results, further molecular investigations took place, and patients 15 and 16 raised positive diagnostic results. In patient 16 the deletion including exons 8 and 9 of the NARS2 gene could not be detected and in patient 15 the homozygous intronic variant was not filtered out during rWES variant calling due to poor sample quality, which was subsequently confirmed as a false negative.

In summary, our findings demonstrate the clinical, social, and economic impact of trio rWES with transcriptome analysis in NICUs, enabling rapid and accurate genetic diagnoses. This tool improves patient management, reduces hospital stays and costs, and offers crucial psychological support for families. Economic analysis highlights its sustainability and benefits for healthcare planning. The progressive incorporation of effective and sustainable innovations in NICUs should be supported by comprehensive, long-term studies, and further research is needed.

## Supplementary Information

Below is the link to the electronic supplementary material.

ESM 1 Whole-exome sequencing in neonates with suspected genetic disease.Patient inclusion flowchart. (PNG 54 KB)

Supplementary file1 (TIFF 7801 KB)

Supplementary file2 (PDF 47 KB)

## Data Availability

The datasets generated during and/or analysed during the current study are available from the corresponding author on reasonable request.

## Abbreviations

ACMG: American College of Medical Genetics and Genomics
COXPD3: Combined oxidative phosphorylation deficiency 3
CNVs: Copy number variations
DEGs: Differentially expressed genes
HAM-A: Hamilton Anxiety Rating Scale
HPO: Human Phenotype Ontology
HSCT: Hematopoietic stem cell transplantation
ICER: Incremental cost-effectiveness ratio
LP: Likely pathogenic
NICUs: Neonatal intensive care units
NHS: National Health System
P: Pathogenic
rWES: Rapid whole-exome sequencing
SD: Standard deviation
UND: Undiagnosed
VUS: Variants of uncertain significance
VCF: Variant call format
WES: Whole-exome sequencing
