# Supplementary figures and images for: Transforming NICU care: rapid WES and transcriptomics—validation, social impact, and cost analysis
Source: Eur J Pediatr. 2025 Jun 27;184(7):453. doi: 10.1007/s00431-025-06225-2 (PMC12205022; doi:10.1007/s00431-025-06225-2)

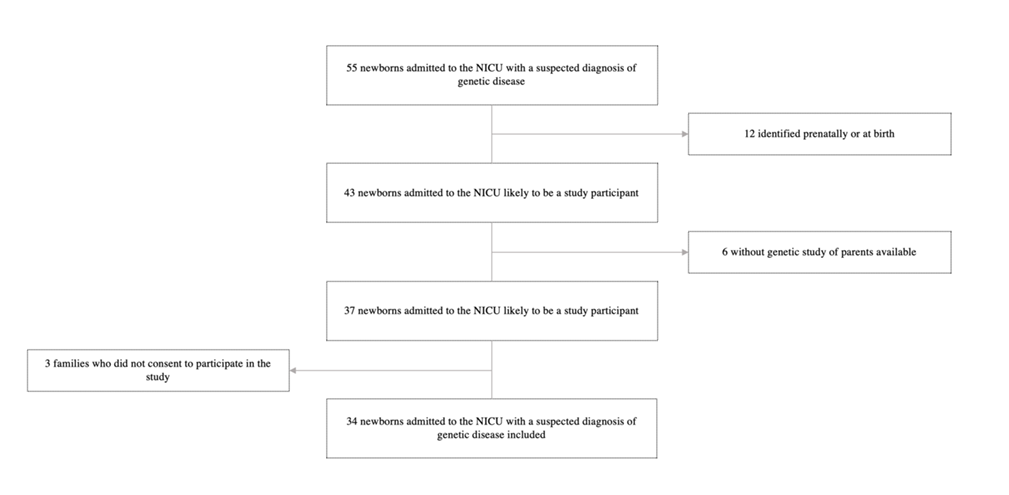

Supplement: Supplementary file 1 — Whole-exome sequencing in neonates with suspected genetic disease.Patient inclusion flowchart. (PNG 54 KB) [file 431_2025_6225_Fig4_ESM.png]

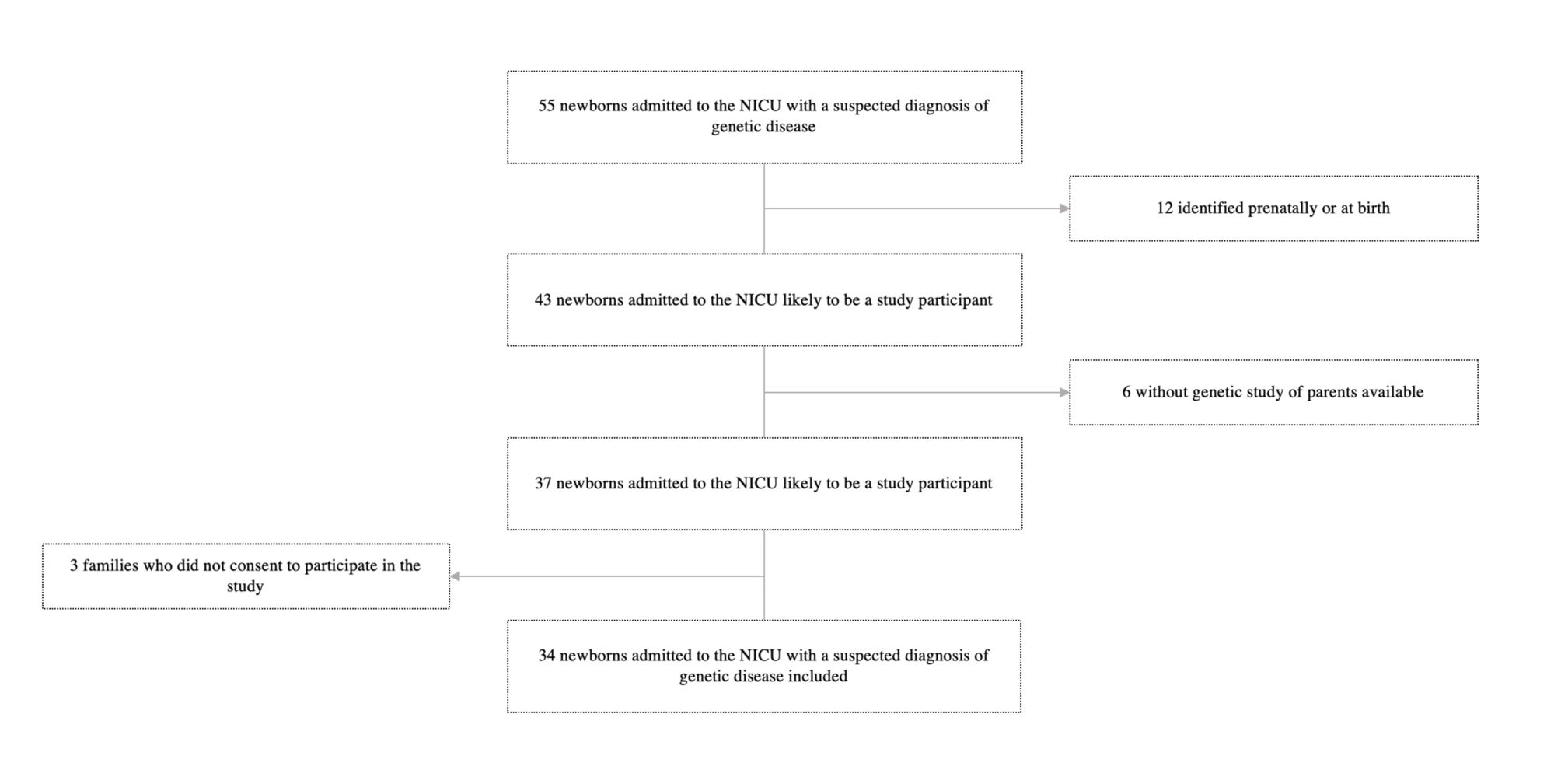

Supplement: Supplementary file 2 — Supplementary file1 (TIFF 7801 KB) [file 431_2025_6225_MOESM1_ESM.tiff]
