# Supplementary material for: Transforming NICU care: rapid WES and transcriptomics—validation, social impact, and cost analysis
Source: Eur J Pediatr. 2025 Jun 27;184(7):453. doi: 10.1007/s00431-025-06225-2 (PMC12205022; doi:10.1007/s00431-025-06225-2)
Supplement: Supplementary file 3 — Supplementary file2 (PDF 47 KB) [file 431_2025_6225_MOESM2_ESM.pdf]

Online Resource 2: Cost-effectiveness according to probability of mortality. Cost-effectiveness for the detected disorders was measured as the probability of mortality in early versus late diagnosis scenarios.

	% Mortality with late diagnosis	% Mortality with early diagnosis
Niemann-Pick type C	73%º	14%
CHARGE syndrome	30%	9%
Polycystic kidney disease with or without liver disease	50%	15%
Familial benign neonatal epilepsy	20%	4%
Wolman disease	100%	40%
Transient infantile liver failure	45%	11%
Combined oxidative phosphorylation deficiency	100%	26%
